# Validation of risk prediction models for sentinel lymph node metastasis in melanoma in a high UV index region

**DOI:** 10.1016/j.jdin.2025.04.012

**Published:** 2025-06-06

**Authors:** Sheyda Mesgarzadeh, Rosemond S. Amamoo, Geethika Ameneni, Amanda H. Gong, Oluwayemisi O. Ayoade, Delaney B. Stratton, Emile Latour, Wesley Yu, Clara Curiel-Lewandrowski, Ivo Abraham, Mohammad Fazel

**Affiliations:** a University of Arizona College of Medicine Tucson, Tucson, Arizona; b University of Arizona Mel and Enid Zuckerman College of Public Health, Tucson, Arizona; c Center for Health Outcomes and Pharmacoeconomic Research, R. Ken Coit College of Pharmacy, University of Arizona, Tucson, Arizona; d Division of Dermatology, University of Arizona, Tucson, Arizona; e Biostatistics Shared Resource, Knight Cancer Institute, Oregon Health & Science University, Portland, Oregon; f Department of Dermatology, Oregon Health & Science University, Portland, Oregon

**Keywords:** melanoma, metastasis, nomogram, risk calculators, risk factors, risk prediction, sentinel lymph node, sentinel lymph node biopsy

## Abstract

**Background:**

Risk prediction models may refine individualized selection for sentinel lymph node biopsy (SLNB) in melanoma.

**Objective:**

To evaluate the statistical accuracy and clinical utility of nomograms by the Melanoma Institute of Australia (MIA), Memorial Sloan Kettering Cancer Center (MSKCC), and University of Colorado in a Southern Arizona population.

**Methods:**

In this prognostic validation, statistical accuracy was assessed through discrimination, measured with receiver operating characteristic curves and calibration plots. Clinical utility was evaluated via decision curve analysis to determine the net benefit and number of net avoidable interventions achieved with nomogram use.

**Results:**

Among 712 melanoma cases included, model discrimination was highest for the MIA nomogram (C-statistic = 0.753; 95% confidence interval = 0.694-0.812), followed by MSKCC (0.729[0.671-0.787]), and University of Colorado (0.601[0.405-0.793]). The MIA and MSKCC nomograms were well-calibrated across clinically relevant risk thresholds. All nomograms achieved a net benefit and net reduction in avoidable SLNBs for risk thresholds ≥5%. There was minimal to no reduction in unnecessary interventions at age extremes (<50 and ≥ 80 years old) for specific risk strata and nomograms.

**Limitations:**

This a 5-year retrospective study.

**Conclusions:**

These nomograms can be used to support SLNB decision-making in this population but necessitate caution in patients at age extremes when used to reduce avoidable interventions.

**Capsule Summary**

• Risk prediction models can help refine individualized risk assessment for nodal involvement and improve selection for sentinel lymph node biopsy in patients diagnosed with melanoma.
• In a Southern Arizona population, 3 clinicopathologic nomograms demonstrate clinical validity but require caution in extremes of age when used to reduce interventions.

## Introduction

In 2025, it is estimated 104,960 new cases of cutaneous melanomas in the United States, with 8430 deaths projected.^1^ In Arizona, a state with predominantly sunny days and a high UV index, approximately 3790 new cases of melanoma are anticipated in 2025. The Arizona Cancer Registry reported a 7.3% increase in the rate of new invasive melanomas between 2017 and 2021.^2^ Accurate patient risk stratification is vital to facilitate timely and appropriate management for melanoma patients.

Patients with invasive cutaneous melanoma may benefit from sentinel lymph node biopsy (SLNB) to evaluate node status, a critical prognostic indicator informing nodal staging, adjuvant therapy eligibility, and surveillance practices.^3^^,^^4^ The National Comprehensive Cancer Network guideline recommendations for identifying patients eligible for SLNB is based on their predicted probability of having a positive sentinel lymph node (SLN).^5^ Per National Comprehensive Cancer Network guidelines, SLNB is generally not recommended for T1a melanoma without adverse features as these are considered to have a <5% risk of SLN metastasis. Patients with T1b melanoma or T1a melanoma with Breslow depth >0.5 mm and other adverse features (eg, age 42, head/neck location, lymphovascular invasion, and/or mitotic rate ≥ 2/mm^2^) are associated with 5% to 10% risk of SLN metastasis and recommended for discussion and consideration of SLNB. Multiple adverse features may confer an additive effect on overall increased risk. Patients with >10% risk, classified as stage T2a and higher, should be offered SLNB.^5^ Integrating personalized patient melanoma characteristics with use of clinicopathologic nomograms may enhance individualized risk assessment and more accurately capture the complex interplay of clinicopathologic variables in the pathogenesis of metastatic disease.^6^, ^7^, ^8^, ^9^ Yet, the generalizability of these tools across diverse geographic populations should not be assumed as differences in how risk factors prevalent among unique populations are represented in prediction tools may limit generalizability.

Nomograms developed by the Melanoma Institute of Australia (MIA), Memorial Sloan Kettering Cancer Center (MSKCC), and the University of Colorado (UCol) provide patient risk prediction for SLN metastasis using clinicopathologic data^7^, ^8^, ^9^ and have been externally validated at national^10^, ^11^, ^12^ and regional levels.^13^, ^14^, ^15^, ^16^ These nomograms show comparable discrimination accuracy but demonstrate mixed results on calibration analysis with evidence of both overestimation and underestimation. When analyzed for risk thresholds of clinical interest (eg, <5%, 5% to 10%, >10%), none have demonstrated cross-population superiority in reducing the number of SLNB performed without missing a positive SLN^12^^,^^15^ or net benefit compared to SLNB for all patients.^12^^,^^13^

This study aimed to investigate the clinical utility of 3 nomograms incorporating clinicopathologic risk factors to ascertain the risk of SLN metastasis in patients treated at the University of Arizona Cancer Center, a region with substantial exposure to UV radiation.

## Methods

### Study design

Nomogram prediction model algorithms were obtained from previously published validation studies.^9^^,^^10^^,^^15^ Patients diagnosed with primary cutaneous invasive melanoma between January 2018 and December 2022 who underwent SLNB were eligible for inclusion. Participants were required to have complete data for all variables specified by at least 1 of the nomograms. Data were collected from the University of Arizona Cutaneous Oncology Program Melanoma Registry, an institutionally hosted retrospective melanoma registry. Exclusion criteria were <18 years of age at time of diagnosis, signs, or symptoms of metastatic disease (palpable lymph nodes, visible in-transit metastasis) before surgical intervention, or neoadjuvant therapy. The University of Arizona IRB approved this study.

### Statistical analysis

Characteristics of patients and their tumors were summarized using counts, percentages, median values, and interquartile ranges. Discriminatory performance was evaluated by plotting the receiver operating characteristic curves, estimating the area under the curve (AUC), and calculating the C-statistic with 95% confidence intervals.

The positive predictive value, negative predictive value (NPV), sensitivity, specificity, overall SLNB reduction rate, net benefit, and net avoidable interventions were calculated at different threshold probabilities (2.5%, 5%, 10%). We compared the observed proportions of positive SLNBs with the expected probability of the MIA and MSKCC models using logistic and flexible (Loess) calibration plots, with the calibration curve for an ideal predictor having an intercept of 0 and a slope of 1.

The clinical net benefit of the models was assessed using decision curve analysis and calculated as the number of patients correctly predicted to have nodal metastasis (true positives) minus the weighted number of patients falsely predicted to have nodal metastasis (false positives) across risk thresholds. A model’s ability to identify patients who will benefit from intervention at a particular risk threshold while reducing the net number of unnecessary interventions was calculated as the net interventions avoided. The overall SLNB reduction rate was calculated as the percentage of patients recognized by the model to avoid SLNB, that is, patients below the specific risk threshold. Analyses were performed using SAS Studio and R 4.4.0.

## Results

### Patient characteristics

A total of 702 patients met criteria for inclusion. Ten patients were diagnosed with more than 1 melanoma, corresponding to 712 treated melanomas. Patients with multiple melanomas were included if their tumors met the following criteria: distinct primary melanomas, identified at separate times or locations, and determined not to be a metastasis, recurrence, or field cancerization. In total, 87 melanomas (12.2%) were SLN-positive with a median patient age (IQR) of 65 (52-75) years compared to the 625 (87.8%) SLN-negative melanomas with a median patient age of 70 (62-77) years (Table I; see Supplementary Table I, available via Mendeley at https://data.mendeley.com/datasets/2fcp9rdr9z/1 for melanomas with missing data). Among all melanomas, 461 (65%) were found in males, 643 (90%) in White, and 681 (96%) in non-Hispanic patients. The majority of tumors were on extremities (41%, *n* = 295). Superficial spreading was the most common subtype (50%, *n* = 356, *P* < .006). SLN-positive compared to node-negative melanomas had a significantly greater Breslow depth [2.1 (1.3-3.4) mm vs 1.3 (0.9-2.1) mm, *P* < .0001], mitotic rate [5.0 (2.0-10.0) mm^2^ vs 2.0 (1.0-5.0) mm,^2^ *P* < .0001], ulceration [59% vs 26%, *P* < .0001)], and presence of lymphovascular invasion [10% vs 2%, *P* < .0017)]. Additional clinicopathologic data are provided in Table I.

**Table I:** Patient characteristics

	Sentinel lymph node biopsy result			
Variable	Overall *N* = 712	Negative *N* = 625	Positive *N* = 87	*P* value
Age, y, median (IQR)	70.0 (61.1-77.0)	70.0 (62.0-77.0)	65.0 (52.0-75.0)	<.0001
<50	73 (10.3)	56 (9.0)	17 (19.5)	
50-59	79 (11.1)	65 (10.4)	14 (16.1)	
60-69	198 (27.8)	176 (28.2)	22 (25.3)	
70-79	248 (34.8)	225 (36.0)	23 (26.4)	
>=80	114 (16.0)	103 (16.5)	11 (12.6)	
Sex, *n* (%)				.9370
Male	461 (64.8)	405 (64.8)	56 (64.4)	
Female	251 (35.3)	220 (35.2)	31 (35.6)	
Race, *n* (%)				.0616
White	643 (90.3)	560 (89.6)	83 (95.4)	
Black	1 (0.1)	1 (0.2)	0 (0.0)	
Asian	1 (0.1)	0 (0.0)	1 (1.2)	
American Indian/Alaskan native	1 (0.1)	1 (0.2)	0 (0.0)	
Native Hawaiian/Pacific Islander	0 (0.0)	0 (0.0)	0 (0.0)	
Two or more races	25 (3.5)	23 (3.7)	2 (2.3)	
Other	22 (3.1)	21 (3.4)	1 (1.2)	
Unknown	19 (2.7)	19 (3.0)	0 (0.0)	
Ethnicity, *n* (%)				.3220
Hispanic or Latin American	25 (3.5)	20 (3.2)	5 (5.8)	
Non-Hispanic	681 (95.7)	599 (95.8)	82 (94.3)	
Unknown	6 (0.8)	6 (1.0)	0 (0.0)	
Tumor thickness, mm, median (IQR)	1.4 (0.9-2.4)	1.3 (0.9-2.1)	2.1 (1.3-3.4)	<.0001
Mitotic rate, mitosis/mm2, median (IQR)	2.0 (1.0-6.0)	2.0 (1.0-5.0)	5.0 (2.0-10.0)	<.0001
Clark level, *n* (%)				
I	2 (0.3)	2 (0.4)	0 (0.0)	
II	3 (0.5)	3 (0.5)	0 (0.0)	
III	35 (5.4)	32 (5.6)	3 (3.9)	
IV	589 (91.0)	517 (90.9)	72 (92.3)	
V	15 (2.3)	14 (2.5)	1 (1.3)	
Not evaluable	3 (0.5)	1 (0.2)	2 (2.6)	.0766
Ulcerated, *n* (%)				<.0001
Absent/not identified	493 (70.0)	458 (74.1)	35 (40.7)	
Present	210 (29.8)	159 (25.7)	51 (59.3)	
Not evaluable	1 (0.1)	1 (0.2)	0 (0.0)	
Lymphovascular invasion present, *n* (%)	22 (3.2)	14 (2.3)	8 (9.5)	.0017
Site, *n* (%)				.1055
Extremity	295 (41.4)	257 (41.1)	38 (43.7)	
Head and neck	195 (27.4)	179 (28.6)	16 (18.4)	
Trunk	222 (31.2)	189 (30.2)	33 (37.9)	
Subtype, *n* (%)				.0059
Acral	21 (3.0)	26 (4.2)	4 (4.6)	
Desmoplastic	27 (3.8)	66 (10.6)	1 (1.2)	
Lentigo maligna	67 (9.5)	130 (20.9)	1 (1.2)	
Nodular	161 (22.7)	314 (50.6)	31 (35.6)	
Superficial spreading	356 (50.3)	17 (2.7)	42 (48.3)	
AJCC 8th edition T category, *n* (%)				<.0001
Tis	2 (0.3)	2 (0.3)	0 (0.0)	
T1a	52 (7.3)	51 (8.2)	1 (1.2)	
T1b	171 (24.1)	160 (25.6)	11 (12.8)	
T2	275 (38.7)	246 (39.4)	29 (33.7)	
T3	132 (18.6)	105 (16.8)	27 (31.4)	
T4	78 (11.0)	60 (9.6)	18 (20.3)	
Additional clinicopathologic variables				
Tumor infiltrating lymphocytes, *n* (%)				.691
Brisk	154 (23.7)	137 (24.0)	17 (22.1)	
Nonbrisk	440 (67.8)	388 (67.8)	52 (67.5)	
Absent	41 (6.3)	34 (5.9)	7 (9.1)	
Growth pattern, *n* (%)				.216
Radial	190 (26.7)	172 (24.2)	18 (2.5)	
Vertical	549 (77.1)	484 (68.0)	65 (9.1)	
Necrosis, *n* (%)	22 (7.5)	14 (5.7)	8 (16.7)	.002
Regression, *n* (%)	177 (25.8)	158 (26.3)	19 (22.4)	.545
Microsatellites, *n* (%)	8 (1.6)	5 (1.2)	3 (4.7)	.098
Perineural invasion, *n* (%)	17 (2.5)	17 (2.9)	0 (0.0)	.204
Associated nevus, *n* (%)	74 (12.5)	65 (12.5)	9 (13.0)	.967

Significance was evaluated by *t*-test for continuous variables and chi-square for categorical variables.

### Model discrimination

**Fig 1:**
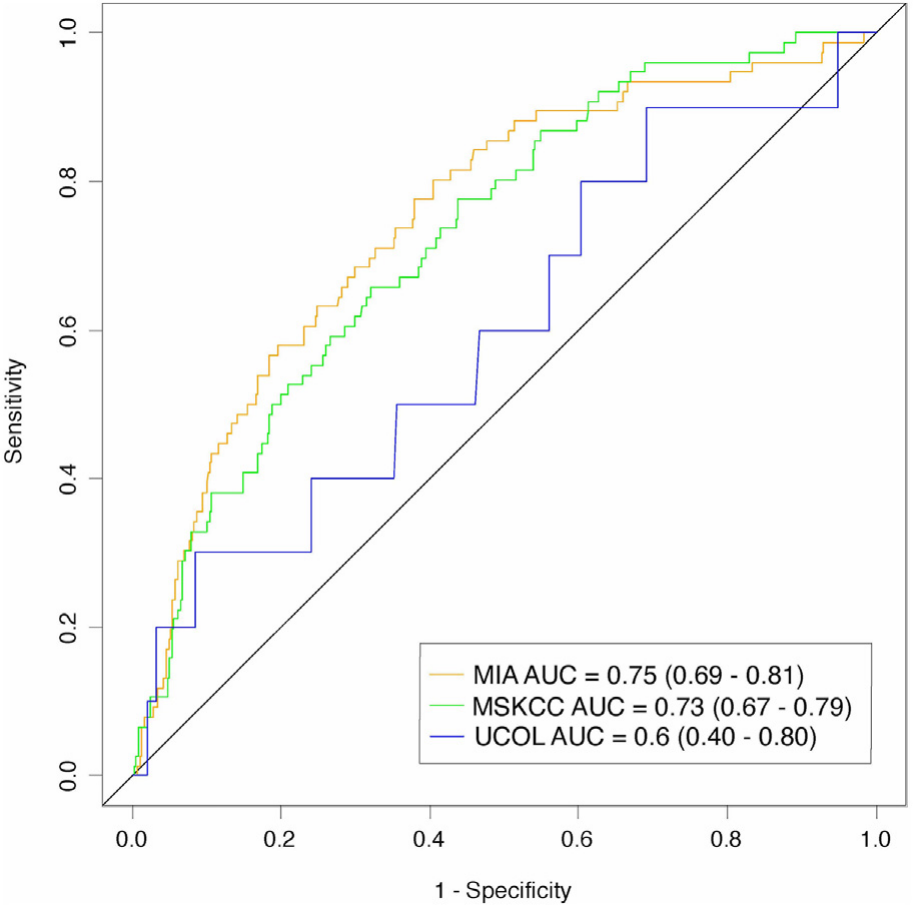
Receiver operating characteristic curves of MIA, MSKCC, and UCol nomograms. *MIA*, Melanoma Institute of Australia; *MSKCC*, Memorial Sloan Kettering Cancer Center; *UCol*, University of Colorado.

The MIA nomogram demonstrated greater discriminatory performance [AUC = 0.753; 95% CI = 0.694-0.812)] over the MSKCC nomogram [AUC = 0.729; 95% CI = 0.671-0.786)], though with overlapping confidence intervals suggesting no statistically significant differences. The UCol nomogram performed nominally lower than both [AUC = 0.601; 95% CI = 0.405-0.796)] (Fig 1). In discrimination subanalyses (Supplementary Table II, available via Mendeley at https://data.mendeley.com/datasets/2fcp9rdr9z/1), restricting analyses to data meeting both MIA and MSKCC inclusion criteria minimally decreased model performance. When stratified by tumor stage, the MSKCC nomogram demonstrated the greatest discrimination for T1 melanomas, whereas the MIA nomogram did so for stage T2a, T2b, and T3a melanomas (Supplementary Table II, available via Mendeley at https://data.mendeley.com/datasets/2fcp9rdr9z/1). When stratified by age, the MSKCC nomogram showed minimally greater discrimination for the 60-69 age group, while the MIA nomogram showed greater discrimination in other age groups (Supplementary Table III, available via Mendeley at https://data.mendeley.com/datasets/2fcp9rdr9z/1). In all subanalyses, overlapping confidence intervals suggest statistically nonsignificant results.

### Calibration and model performance

**Fig 2:**
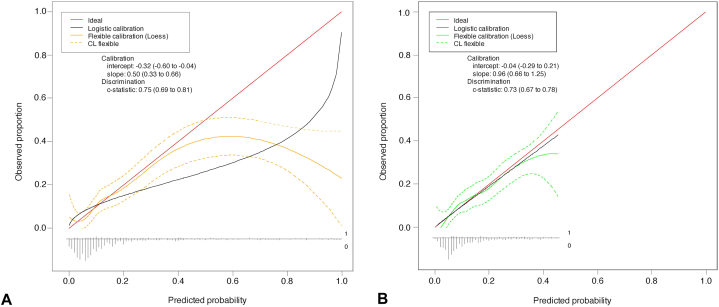
**A** and **B,** Calibration plots of MIA and MSKCC nomograms. *MIA*, Melanoma Institute of Australia; *MSKCC*, Memorial Sloan Kettering Cancer Center.

The MIA nomogram revealed moderate calibration, underestimating predicted probabilities at lower risk thresholds and shifting towards overestimation at higher thresholds (intercept = −0.32, slope = 0.50) (Fig 2, *A*). In comparison, the MSKCC nomogram demonstrated the best calibration across all predicted risk probabilities (intercept = −0.04; slope = 0.96) (Fig 2, *B*). Limited sample size did not allow calibration analysis for the UCol nomogram.

At the 5% risk threshold, MIA, MKSCC, and UCol nomograms demonstrated high NPVs. The MIA and MSKCC nomograms provided greater sensitivity compared to the higher specificity of the UCol nomogram. At the 10% risk threshold, there was a decrease in NPVs and sensitivities alongside an increase in specificity across all nomograms (Table II).

**Table II:** Metrics of model performance at guideline-endorsed thresholds

Threshold, model, age stratification	NPV, %	PPV, %	Sensitivity, %	Specificity, %	Overall SLNB reduction rate∗,%	Net benefit of the model†	Net benefit SLNB for all†	Net benefit compared to SLNB for all†	Net avoidable interventions‡
5%									
MIA	96.7	15.5	93.4	27.7	25.08	0.083	0.079	0.004	8.69
<50	100	23.7	100	2.2		0.194			1.67
50-59	100	18.2	100	8.5		0.129			7.04
60-69	96.9	13.7	95.0	20.5		0.074			7.02
70-79	97.3	13.0	90.0	37.6		0.054			16.36
>=80	95.0	14.8	80.0	45.2		0.059			0
MSKCC	97.4	14.0	96.1	20.1	18.18	0.077	0.073	0.004	8.78
<50	90	24.0	92.3	19.2		0.167			−16.67
50-59	100	19.6	100	13.8		0.134			11.43
60-69	100	13.5	100	16.2		0.076			14.29
70-79	100	11.9	100	18.9		0.060			17.04
>=80	94.1	8.7	75.0	33.7		0.026			−5.83
Ucol	95.7	8.1	30.0	82.2	81.59	0.006	−0.000	0.006	11.94
10%									
MIA	95.0	21.6	77.6	59.9	55.25	0.058	0.027	0.031	27.38
<50	92.3	27.7	92.9	26.1		0.154			5
50-59	95.8	23.4	91.7	39.0		0.099			19.72
60-69	94.5	18.8	75.0	57.0		0.045			23.98
70-79	95.1	18.6	65.0	70.6		0.031			34.58
>=80	95.4	24.1	70.0	73.8		0.048			37.23
MSKCC	93.9	19.3	71.1	59.8	56.11	0.045	0.021	0.024	21.63
<50	87.9	33.3	69.2	61.7		0.117			−11.67
50-59	90.9	24.3	75.0	51.7		0.084			4.29
60-69	93.7	17.2	71.4	55.3		0.038			19.23
70-79	94.9	17.2	68.2	64.2		0.031			29.60
>=80	96.7	14.3	75.0	72.1		0.019			39.81
Ucol	95.0	0	0	98.4	98.51	−0.002	−0.056	0.054	48.76
2.5%									
UCol	97.0	5.90	80.0	33.5	32.86	0.024	0.025	−0.001	−6.97

*MIA*, Melanoma Institute of Australia; *MSKCC*, Memorial Sloan Kettering Cancer Center; *NPV*, negative predictive value; *PPV*, positive predictive value; *SLNB*, sentinel lymph node biopsy; *UCol*, University of Colorado.

∗ The overall SLNB reduction rate is the proportion of the total patients predicted to have a risk of sentinel lymph node positivity lower than the risk threshold.

† The unit for net benefit is the number of true positives.

‡ Net avoidable interventions is per 100 patients compared to SLNB for all patients. The unit for net avoidable interventions is the number of true negatives.

### Decision curve analysis

**Fig 3:**
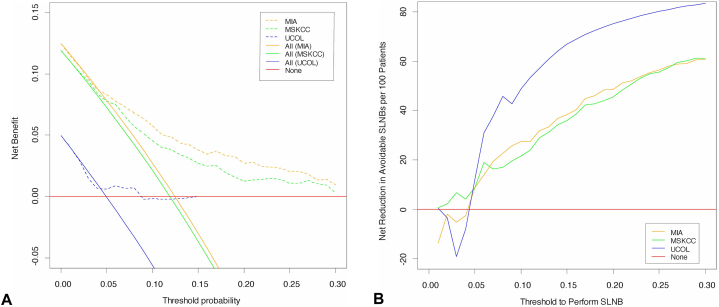
Decision curve analysis of Melanoma Institute of Australia (MIA) and Memorial Sloan Kettering Cancer Center (MSKCC) nomograms. **A,** Net benefit of MIA and MSK nomograms compared to treat all approach. **B,** Net reduction in avoidable sentinel lymph node biopsy (SLNB).

All nomograms provide a positive net benefit compared to the treat-all approach for risk thresholds ≥5%. The net benefit is greater at the 10% risk threshold (MIA 0.031, MSKCC 0.024, UCol 0.054) compared to the 5% threshold (MIA 0.004, MSKCC 0.004, UCol 0.006) (Fig 3, *A*, Table II). At the 5% risk threshold, the MIA, MSKCC, and UCol nomograms misclassified, respectively, 5, 3, and 7 patients with true metastatic disease as not having metastatic involvement (false negative). At the 10% risk threshold, respectively, 17, 22, and 10 patients were misclassified (Supplementary Table IV, available via Mendeley at https://data.mendeley.com/datasets/2fcp9rdr9z/1).

Across 5% and 10% risk thresholds, all nomograms provided a reduction in the overall rate of SLNBs performed (Table II). The net avoidable intervention analysis evaluated the number of correctly avoided SLNBs per 100 patients without missing a positive SLN (Fig 3, *B*, Table II). The number of SLNBs correctly avoided using the MIA, MSKCC, and UCol nomograms were 9, 9, and 12 SLNBs, respectively, at the 5% risk threshold, and 27, 22, and 49 SLNBs, respectively, at the 10% risk threshold.

The effect of net avoidable interventions reduces to zero or negative at certain age strata and risk thresholds suggesting a loss of effect (Table II). At the 5% risk threshold, the MSKCC and MIA nomograms provide minimal to no reduction in the net avoidable SLNBs for patients at extremes of age (<50 years and ≥80 years) and similarly at the 10% threshold for patients <50 years using the MSKCC nomogram. Interpretation of the UCol nomogram results is limited by the low 10 node-positive melanomas compared to the 191 node-negative melanomas.

## Discussion

This report presents the results of a single-institution validation of 3 nomograms developed to predict the risk of SLN metastasis in melanoma. The MIA and MSKCC nomograms demonstrate comparable discriminatory performance across our population, consistent with previous validation efforts.^10^, ^11^, ^12^, ^13^^,^^15^ The smaller dataset for the UCol nomogram, limited to thin melanomas (Breslow 0.5-1.0 mm), may have contributed to its overall poorer discrimination. Calibration analysis of the MIA and MSKCC nomograms showed strong agreement between the predicted probability and the observed proportion of positive SLNB. The MSKCC nomogram demonstrates near-ideal calibration, whereas the MIA nomogram exhibits overestimation of predicted risk with increasing risk thresholds. The clinical significance of this overestimation at higher risk thresholds is limited, as predicted risks above 10% generally undergo SLNB despite nomogram-calculated risk.^5^

The purpose of measures of model performance is to assess the statistical accuracy of predictive tools. Decision curve analysis adds the important dimension of translational benefit in clinical practice by weighing the benefit of accurately identifying disease states or avoiding unnecessary intervention against incorrectly classifying diseases that may otherwise result in undue harm. All 3 models provide a positive net benefit compared to SLNB for all at risk thresholds ≥5%. However, this effect is minimal at the 5% threshold, at which previous studies have similarly demonstrated inconsistent to no benefit across low to intermediate-risk thresholds (<10%) due to the false positives outweighing the true positives identified by the nomograms.^12^^,^^13^^,^^17^

All models offer a reduction in the number of unnecessary SLNBs performed per 100 patients without incorrectly missing patients with nodal disease at threshold probabilities ≥5%. When stratified by age, patients at extremes of age (<50 years using the MSKCC nomogram at the 5% and 10% risk threshold, and ≥80 years using the MIA and MSKCC nomograms at the 10% risk threshold) may not benefit to the same degree from the use of these tools as a resource to reduce the number of unnecessary interventions as seen by the reduction in ability to avoid unnecessary intervention.

Variability in nomogram performance across validation studies may arise from differences in study populations. An MIA nomogram validation by Maddineni et al at Stanford Medical Center reported an overall underprediction of risk and lack of benefit compared to the treat-all approach at lower risk thresholds. The authors identified differences in tumor characteristics among their population compared to the original MIA development cohort, including thinner melanomas, lower mitotic rates, and differing proportions of melanoma subtypes represented.^13^ Notably, our Southern Arizona cohort demonstrates an older median age at diagnosis compared to development^7^, ^8^, ^9^ and external validation study cohorts,^10^^,^^11^^,^^13^, ^14^, ^15^, ^16^ and a lower sentinel node positivity rate of 12.2%.^7^^,^^8^ Aside from variability in patient population, contrasting methodology and lack of assumed variables in the presented data are contributing factors in the differences in results. While these differences cannot entirely account for the observed variability in nomogram performance, they highlight the need for further investigation. A study utilizing data from the Swedish Melanoma Registry found that the MIA and MSKCC nomograms provided the greatest net benefit for T2 melanomas and net avoidable interventions at the 10% risk threshold. However, modifications to MIA nomogram variable inputs were necessary based on Swedish Melanoma Registry data availability–lymphovascular invasion was not included and mitotic rates were reported as present or absent.^12^ These are optional inputs in the MIA nomogram. In contrast, a validation effort from MSKCC that did not require variable modifications found no clinically relevant reduction in net avoidable interventions with the MIA and MSKCC nomograms.^15^ Consideration of nomogram modeling techniques is critical when interpreting study results and evaluating their translatability across populations.

As risk prediction tools become increasingly integrated into clinical practice, it is essential to prioritize accuracy and practicality for routine use. While genetic expression profiling tools show promise in contributing to the decision-making for SLNB,^18^^,^^19^ their utility in clinical settings is currently unclear.^19^, ^20^, ^21^, ^22^ Further research is needed to assess the cost-effectiveness of these approaches and compare the performance of clinicopathologic and genetic expression profiling-based tools directly.^23^

The strengths of this study include the use of a reliable, comprehensive single-institutional database that captures all patients who underwent SLNB. All referred cases are evaluated by internal pathologists, ensuring consistency in interpretation and reporting. No modifications to variable inputs or data imputations for missing variables were needed. To verify that our patient cohort reflects our communities, we confirmed that the greater median age, race, and ethnic distributions observed in our patient population correlate with state-level distributions as reported by the Arizona Cancer Registry.

This study is limited by the small sample size and restricted years under evaluation; however, this allowed for all patient staging under the same AJCC edition. These nomograms are intended for use before SLNB, when only preliminary data based on the diagnostic biopsy may be available. Final T-staging is determined after wide local excision (WLE), which is performed concurrently with SLNB at our institution. Thus, SLN metastasis risk stratification occurs before complete lesion characterization and is subject to change pending WLE results. Preliminary analysis showed that only 5% of cases were upgraded to a higher T stage after WLE, and it was decided not to use WLE data to remain consistent with our intent to validate these tools per their intended clinical use in our practice.

## Conclusion

The MIA, MSKCC, and UCol nomograms demonstrate appropriate statistical accuracy and offer clinical utility in providing a net benefit and reducing avoidable interventions in this unique, high UV index region in Southern Arizona. This population is notable for a greater age at melanoma diagnosis and treatment, with SLNB having a lower sentinel node positivity rate. Further investigation of these tools revealed a minimal to no reduction in unnecessary interventions at extremes of age (<50 and ≥ 80 years old) for specific risk strata and nomograms, suggesting caution in use for these patients. Our findings corroborate the uncertainty in nomogram generalizability observed in other validation studies of regional populations, emphasizing the need to evaluate nomogram performance with population-specific characteristics.^13^^,^^15^ A statistically accurate test does not guarantee clinical appropriateness, and future validation studies should prioritize investigating the clinical utility of these tools. Ultimately, these risk prediction nomograms are a valuable resource to be used alongside clinical judgment to improve patient care and optimize outcomes.

## Conflicts of interest

None disclosed.
